# Uncertainties in the projection of species distributions related to general circulation models

**DOI:** 10.1002/ece3.1411

**Published:** 2015-02-13

**Authors:** Eric Goberville, Grégory Beaugrand, Nina-Coralie Hautekèete, Yves Piquot, Christophe Luczak

**Affiliations:** 1 Laboratoire de Génétique et Evolution des Populations Végétales, UMR 8198 GEPV, Université Lille 1 – Sciences et Technologies (USTL)F-59655, Villeneuve d'Ascq, France; 2 Laboratoire d'Océanologie et de Géosciences, UMR 8187 LOG, Université Lille 1 – Sciences et Technologies (USTL)28 Avenue Foch, F-62930, Wimereux, France; 3 Laboratoire d'Océanologie et de Géosciences, CNRS, UMR 8187 LOG28 Avenue Foch, F-62930, Wimereux, France; 4 The Laboratory, Sir Alister Hardy Foundation for Ocean Science (SAHFOS)Citadel Hill, Plymouth, PL1 2PB, UK; 5 Université d'Artois, ESPE, Centre de Gravelines40 rue Victor Hugo – BP 129, 59820, Gravelines, France

**Keywords:** Biogeography, climate change, ecological niche modeling, global change models, species distribution projections, uncertainties

## Abstract

Ecological Niche Models (ENMs) are increasingly used by ecologists to project species potential future distribution. However, the application of such models may be challenging, and some caveats have already been identified. While studies have generally shown that projections may be sensitive to the ENM applied or the emission scenario, to name just a few, the sensitivity of ENM-based scenarios to General Circulation Models (GCMs) has been often underappreciated. Here, using a multi-GCM and multi-emission scenario approach, we evaluated the variability in projected distributions under future climate conditions. We modeled the ecological realized niche (*sensu* Hutchinson) and predicted the baseline distribution of species with contrasting spatial patterns and representative of two major functional groups of European trees: the dwarf birch and the sweet chestnut. Their future distributions were then projected onto future climatic conditions derived from seven GCMs and four emissions scenarios using the new Representative Concentration Pathways (RCPs) developed for the Intergovernmental Panel on Climate Change (IPCC) AR5 report. Uncertainties arising from GCMs and those resulting from emissions scenarios were quantified and compared. Our study reveals that scenarios of future species distribution exhibit broad differences, depending not only on emissions scenarios but also on GCMs. We found that the between-GCM variability was greater than the between-RCP variability for the next decades and both types of variability reached a similar level at the end of this century. Our result highlights that a combined multi-GCM and multi-RCP approach is needed to better consider potential trajectories and uncertainties in future species distributions. In all cases, between-GCM variability increases with the level of warming, and if nothing is done to alleviate global warming, future species spatial distribution may become more and more difficult to anticipate. When future species spatial distributions are examined, we propose to use a large number of GCMs and RCPs to better anticipate potential trajectories and quantify uncertainties.

## Introduction

Over the last few decades, global climate change has caused consistent patterns of phenological and biogeographic shifts in species (Parmesan and Yohe 2003; Körner and Basler 2010). As warming is likely to range between ∽1 and ∽5°C by 2100 (Knutti and Sedlacek 2012), these changes may amplify toward the end of this century (Pereira et al. 2010). Based on the relation between a species and its environment, Ecological Niche Models (ENMs) or Species Distribution Models (SDMs) have been applied extensively to investigate the potential implications of future climate change for species distributions (Peterson 2006; Raybaud et al. 2013). However, it is now well documented that any projection of a future species distribution will have an associated level of uncertainty (Wiens et al. 2009; Beale and Lennon 2012). Identifying and quantifying the sources of uncertainty that affect simulations of future species distributions are therefore a required step for improving the reliability of projections (Beaumont et al. 2007).

Ecological Niche Models are often combined with outputs from General Circulation Models (GCMs) to evaluate potential changes in the range of species as a function of emissions scenarios (Peterson 2006). These scenarios, based on different socioeconomic, technological and environmental trends (Nakicenovic et al. 2000), focus on long-term trends in energy and land use to evaluate the response of the climate system facing to change in greenhouse gases concentrations (Rogelj et al. 2012). However, working with outputs from GCMs does not imply predicting the future, but better assessing uncertainties under a wide range of possible futures (Moss et al. 2010). GCMs do not represent a crystal ball for the future, and concerns exist about their ability to simulate the response of a major mode of global circulation variability to external forcings (Driscoll et al. 2012; IPCC 2013). Current GCMs may diverge for technical or parameterization reasons (e.g., parameterization of natural processes such as ocean mixing and spatial resolution; Intergovernmental Panel on Climate Change 2007). Different GCMs may also simulate feedback processes relating to water vapor or clouds in different ways (Wiens et al. 2009). The outputs of simulated environmental variables from different GCMs may also vary due to diverse downscaling approaches (Timbal 2004). Far from being exhaustive, this list reveals the wide variety and complexity of GCMs. It is thus difficult to identify a modeling algorithm that performs better than another (Martinez-Meyers 2005), and the choice of a GCM may greatly influence the projected distributions of a species (Real et al. 2010). Nevertheless, studies still rarely consider individually several GCMs to take into account this source of uncertainties in species projections (Beaumont et al. 2007; Buisson et al. 2010) and even fewer quantify uncertainties arising from different GCMs compared to those originating from the different trajectories of greenhouse gas concentrations (Real et al. 2010).

Here, we focused on the dwarf birch (*Betula nana*) and sweet chestnut (*Castanea sativa*) for their well-known distribution and for their distinct life histories (Jalas and Suominen 1972; Ohlemüller et al. 2006). These species belong to two major functional groups of European trees (Smith et al. 2001) and are representative of contrasting spatial patterns (Thuiller 2003): a subarctic species common in taiga and montane regions, generally above 300 m (*B. nana*; De Groot et al. 1997) and a temperate species widespread in southern and western Europe (*C. sativa*; Haltofová and Jankovský 2003). They have distinct climatic requirements and have been shown to be sensitive to climate-induced changes (Sturm et al. 2001b; Thuiller 2003). The ecological niche (*sensu* Hutchinson 1957) of both species was modeled using the Non-Parametric Probabilistic Ecological Niche model (NPPEN; Beaugrand et al. 2011) and projected onto a geographical space to map their baseline distributions (1950–2000) in terms of probability of occurrence. Using seven GCMs and four emissions scenarios originating from the new IPCC “Representative Concentration Pathways” (RCPs), we evaluate the influence of climate change on the spatial distribution of these two species from the baseline period to the end of this century. While many studies exhibit substantial differences in the projection of species related to data quality (Franklin 2009), model algorithms (Thuiller 2003), emissions scenarios (Beaugrand et al. 2011) or the choice of predictor variables (Peterson and Cohoon 1999), we reveal that GCMs are also a major source of uncertainties in ENM projections. In addition, we show that the variability related to GCMs magnifies when the intensity of warming increases.

## Materials and Methods

### Observed species distribution

We modeled baseline and future species distributions for two European species: the sweet chestnut (*C. sativa*) and dwarf birch (*B. nana*).

*Castanea sativa* is a deciduous temperate species (Benito Garzon et al., 2008) with relatively high temperature (10–15°C) and moisture demands (mean annual precipitation between 500 and 2500 mm; Krebs et al. 2004). The tree has a rather marked preference for an oceanic climate (Krebs et al. 2004), prefers moderate winters, and requires warm dry summers to ripen their fruit (Howes 1948). The species is tolerant of highly acid and infertile dry sands but averse to calcareous soils (Huxley 1992). Previous studies have revealed the importance of both temperature and precipitation factors for this tree (e.g., Thuiller et al. 2003), which is expected to decrease in productivity under high emissions scenarios (Broadmeadow et al. 2005).

*Betula nana* is a prostrate shrub native to regions with long cold winters and short cool summers (Huxley 1992). Shrubs do not have the same requirements than trees for temperature, and *B. nana* can resist down to 6°C summer temperatures (Thompson et al. 2006). De Groot et al. (1997) mentioned an optimum temperature for photosynthesis of 10–13°C, and annual precipitation across the species range varies from 300 mm in circumpolar regions to 2000 mm in the British Isles. Wind and solar radiation also influence the species distribution (Anderson et al. 1966; De Groot et al. 1997), and snow cover can increase shrub tolerance to extreme cold and wind-induced desiccation. With an increase in temperature, it is expected that *B. nana* biomass will expand in the Arctic region (Euskirchen et al. 2009) with implications on the surface energy balance and the permafrost thaw (Blok et al. 2010).

**Figure 1:**
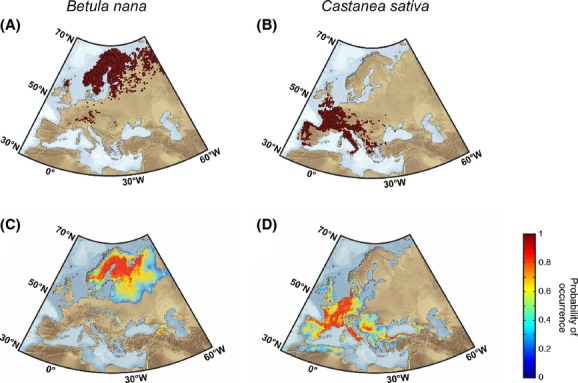
Observed and modeled spatial distributions of *Betula nana* and *Castanea sativa* for the baseline period 1950–2000. Observed spatial distributions (as occurrence) of (A) *Betula nana* and (B) *Castanea sativa* in Europe from the *Atlas Florae Europaeae*. Modeled spatial distributions (as probability of occurrence) of (C) *B. nana* and (D) *C. sativa* calculated from the NPPEN model. Data below 0.273 (*B. nana*) and 0.194 (*C. sativa*) were removed after application of the MDT criterion (see Materials and Methods).

Baseline distributions were obtained from the *Atlas Florae Europaeae* (Jalas and Suominen 1972), which uses 50 × 50-km^2^ grid cells (Fig.1A–B). We only retained occurrence records termed as “certain” by the AFE.

### Environmental data and selection of the climatic parameters

The selection of ecologically relevant variables is a prerequisite to model the ecological niche of a species (Elith and Leathwick 2009; Franklin 2009). Here, environmental data for the period 1950–2000 were retrieved from the WorldClim dataset (Hijmans et al. 2005; http://www.worldclim.org/). Calculated from monthly temperature and precipitation climatologies, these variables reflect spatial variations in annual means, seasonality, and extreme/limiting conditions (Table S1). These environmental variables, appropriate for characterizing terrestrial species range (Roubicek et al. 2010), are closely related to plant and tree physiological limitations (Bartlein et al. 1986; Prentice et al. 1992; Pearman et al. 2008). Owing to interactions of temperature and moisture availability, seasonal variations and extreme climate events could more strongly influenced species distributions than annual means (Bakkenes et al. 2002; Stockwell 2006). Information on climate parameters for the period 1950–2000 was added to each observation of species occurrence by interpolation of each environmental data point from the dataset described above (Beaugrand et al. 2011). Modeled species distributions were then projected back onto the spatial resolution of 0.1° latitude × 0.1° longitude for baseline and future climate.

Multicollinearity among predictors may hamper the analysis of species–environment relationships (Heikkinen et al. 2006) and increase model uncertainties (Stockwell 2006). To model the ecological niche of species, it is important to identify explanatory variables, which mainly influence species spatial distribution (Franklin 2009). Climate predictors were thus screened for multicollinearity before application of the ENM. To do so, we applied the Escoufier procedure (Robert and Escoufier 1976), so-called ‘R_V_-coefficient’ procedure, for variable selection. The R_V_-coefficient is a typical example of a matrix correlation introduced as a measure of similarity between squared symmetric matrices (Escoufier 1973; Robert and Escoufier 1976) and could be considered as a multivariate generalization of the Pearson correlation coefficient (Legendre and Legendre 1998). This coefficient measures the similarity between *h*-dimensional and *i*-dimensional matrices with the same *g* observations. Let **X**_g,h_ be the (*g* × *h*) matrix of *g* observations and *h* descriptors and **Y**_g,i_ be the (*g* × *i*) subset of *g* observations and *i* descriptors of **X**, thereafter termed **Y**_(i)_. The coefficient *R*_V_(**X**,**Y**_(i)_) ranges in the closed interval [0 1] and quantifies the ability of the *i* descriptors of subset **Y** to summarize the whole information of **X**: the closer to 1 the *R*_V_(**X**,**Y**_(i)_) is, the better **Y**_(i)_ is a substitute for **X**. Using a forward stepwise selection of variables, the *k*th variable is introduced to optimize *R*_V_(**X**,**Y**_(k)_) when *k*−1 variables have already been added. No statistical test of the significance of a *R*_V_ value exists (Schlich and Guichard 1989). Therefore, since the magnitude of *R*_V_ value is comparable to that of a squared correlation, a *R*_V_ around 0.95 indicates good similarity between the whole and the reduced dataset (Schlich and Guichard 1989).

For each species, we constructed a matrix of 19 descriptors corresponding to the presence records (1238 observations for *B. nana* and 695 observations for *C. sativa*). Applying the “*R*_V_-coefficient” procedure on each matrix, we calculated two subsets (one for *B. nana* and one for *C. sativa*) with a reduced number of descriptors. When the *R*_V_ value reached a value around 0.95, the adding of climatic parameters was stopped (Fig. S2). We retained the following two sets of environmental factors to evaluate both baseline (1950–2000) and future potential distributions (Table S2): (1) temperature annual range, annual mean temperature, and precipitations of the driest and coldest quarter for *B. nana*; and (2) annual mean temperature, temperature and precipitation seasonality (as standard deviation for temperature and coefficient of variation for precipitation; http://www.worldclim.org/), annual precipitation, and precipitation of the warmest quarter for *C. sativa*. The two sets of parameters, revealing distinct climatic requirements for both species, appear congruent with the factors influencing the ecology and distribution of both species (see paragraph “Observed species distribution”).

### Estimation of the future bioclimatic parameters using data originated from GCMs

While the fourth assessment report of the Intergovernmental Panel on Climate Change (IPCC 2007) was based on the Coupled Model Intercomparison Project3 (CMIP3) and emissions scenarios from the “Special Report on Emissions Scenarios” (SRES), a new set of four trajectories of greenhouse gas concentrations based on the fifth phase of the Coupled Model Intercomparison Project5 (CMIP5; http://cmip-pcmdi.llnl.gov/cmip5/) was designed for the IPCC fifth assessment report (IPCC 2013). The new set of scenarios, called “Representative Concentration Pathways” (RCPs) are labeled according to their specific radiative forcing pathway in 2100 relative to pre-industrial values: RCP2.6, RCP4.5, RCP6.0, and RCP8.5 (Table S3). The emergence of new technologies, recent assumptions about socioeconomic development as well as observations of environmental factors such as land use and land cover change have been considered in this new generation of scenarios (Moss et al. 2010; Rogelj et al. 2012; van Vuuren et al. 2012). The RCPs explicitly explore the impact of different climate policies in addition to the no-climate-policy SRES scenarios (van Vuuren et al. 2011b) and provide an important reference point to investigate the potential implications of climate change on ecosystems (van Vuuren et al. 2011a).

To evaluate the potential future distribution of *B. nana* and *C. sativa*, we used these RCPs emissions scenarios. The outputs of simulated precipitation, and both minimum and maximum temperatures from seven high-resolution General Circulation Models were used in this study (CNRM-CM5, CSIRO-Mk3.6.0, IPSL-CM5A-LR, HadGEM2-ES, MPI-ESM-LR, GISS-E2-R, and CCSM4), with all available RCP scenarios: the low RCP2.6, the medium–low RCP4.5, the medium–high RCP6.0, and the high RCP8.5. Note that no simulation was carried out by the CNRM-CERFAS for the RCP2.6 and RCP6.0 scenarios and by the MPI-M for the RCP6.0 scenario. We selected these GCMs as they have been commonly used in recent studies dealing with the impacts of climate change on biodiversity (e.g., Buisson et al. 2010; Naujokaitis-Lewis et al. 2013; Raybaud et al. 2013), and carefully described (basic information on each GCM is provided in Table S4).

For the period 1950–2100, monthly time series of precipitation, minimum and maximum temperatures for each of the seven GCMs (Table S5) were downloaded from the Earth System Grid Federation portal (ESGF; http://pcmdi9.llnl.gov/esgf-web-fe/). To minimize the effects of possible bias between data used to model baseline distributions and GCM outputs (Huntley et al. 2007), we adopted an approach based on the sum of anomalies (Ramírez-Villega and Jarvis, 2010). For each GCM simulation, the method produces surfaces of changes in precipitation, minimum and maximum temperatures (called “anomalies”) and these surfaces were then added to the data used to model baseline distributions. Following the “delta method” procedure (Ramírez-Villegas and Jarvis 2010; Fig. S5), differences in baselines were neglected for temperatures but considered for precipitation (see equations 4 and 5 in Ramírez-Villegas and Jarvis 2010).

Here, using GCM outputs from 1950 to 2000, we first calculated 252 climatologies (7 GCMs × 3 variables × 12 months) for the baseline period common to the one used to produce the WorldClim dataset (i.e., 1950–2000; Hijmans et al. 2005). Using GCM outputs from 2010 to 2100, we subsequently calculated future climatic conditions in precipitation, minimum and maximum temperatures for eight 20-year periods from 2010 to the end of this century (i.e., 2010–2029, 2020–2039, 2030–2049, 2040–2059, 2050–2069, 2060–2079, 2070–2089, and 2080–2099). The procedure was carried out for each month (12) of each 20-year period (8), all GCMs (7), all emission scenarios (4), and for the three variables, giving a total of 7200 climatologies. For each 20-year period, anomalies in precipitation, minimum and maximum temperatures were calculated for each month (i.e., difference between a given 20-year period and the period 1950–2000) and an interpolation procedure was applied to generate gridded data at the spatial resolution of 0.1° latitude × 0.1° longitude. We used the minimum curvature method from the Spatial and Geometric Analysis toolbox (SaGA; http://puddle.mit.edu/∽glenn/kirill/saga.html). We acknowledge other interpolation methods exist (Wang et al. 2012; Sachindra et al. 2014), but this procedure is known as suitable and computationally efficient to perform downscaling (Beaumont et al. 2007; Huntley et al., 2008). These anomalies were then added to the 1950–2000 WorldClim climatologies, following the “delta method” procedure defined by Ramírez-Villegas and Jarvis (2010). We then generated the 19 bioclimatic variables included in the WorldClim dataset (Table S1) applying the method provided by Ramírez-Villegas and Bueno-Cabrera (2009) and retained the environmental factors previously used to model baseline distributions (Table S2).

### Modelling of the ecological niche of species

We modeled the ecological niche *sensu* Hutchinson (i.e., the combination of the environmental factors required by a species) of *B. nana* and *C. sativa* and projected their spatial distribution using the Non-Parametric Probabilistic Ecological Niche model (NPPEN; Beaugrand et al. 2011), which only requires presence data. The NPPEN model, based on a nonparametric procedure and the Mahalanobis distance (which is independent of the scales of the descriptors; Legendre and Legendre 1998), enables correlations between environmental factors to be taken into account (Ibañez 1981; Farber and Kadmon 2003). This model allows the modelling of the ecological niche of a species and the mapping of its spatial distribution by calculating probabilities of occurrence. As the technique has been fully described by Beaugrand et al. (2011) and applied elsewhere (e.g., Goberville et al. 2011; Lenoir et al. 2011; Rombouts et al. 2012; Chaalali et al. 2013; Raybaud et al. 2013; Beaugrand et al. 2014), we refer the reader to this literature for a more detailed mathematical description and only recall the main steps of calculation. The first step consists in constructing a reference matrix (**Z**_m,p_) with environmental data corresponding to the presence records. However, the reference matrices used to calculate the probability of occurrence of species could be biased toward regions more investigated than others (e.g., easily accessible, surveyed regions…). Such a bias can lead to an over-representation of environmental features (Kramer-Schadt et al. 2013) and to lack of independence between training and test datasets (Veloz 2009). This lack of independence can then influence modelling algorithms and validation procedures when AUC tests are performed (Veloz 2009). To consider this potential bias, we homogenized each reference matrix before the application of the model to (1) eliminate the potential effect of oversampling and (2) remove as far as possible the inaccurate reporting of occurrence records: single observations or cells with missing environmental data were removed from the reference matrix (**Z**_m,p_). In this way, duplicate records were removed by absorption into a single cell (Rombouts et al. 2012). By assigning the same weight to over and/or undersampled regions, this procedure eliminates the influence of one single misreporting (Beaugrand et al. 2011; Lenoir et al., 2013). For each species, a multidimensional matrix was defined, each of the dimension reflecting an environmental factor (4 and 5 dimensions for *B. nana* and *C. sativa*, respectively). A cell of the homogenized matrices could therefore be considered as a class of environmental stratum. Each environmental stratum belonging to the geographical cell (0.1° latitude × 0.1° longitude) in the original data matrix was then compared to the condensed environmental matrix. If the environmental stratum corresponded, we retained one data occurrence. This procedure is similar to the one performed in the programme RASTERIZ included in the GARP modelling system (Stockwell 1999). In a second step, the Mahalanobis generalized distance (Ibañez 1981) is calculated between the observations and the homogenized reference matrix: 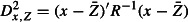
1with *x* the vector of length *p*, representing the values of the environmental data to be tested, ***R***_*p,p*_ the correlation matrix of reference matrix ***Z***_*m,p*_ and 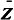 the average environmental condition inferred from ***Z***_*m,p*_. The use of the Mahalanobis distance instead of a classical Euclidian distance presents a double advantage: it enables the correlation between variables to be taken into account (Ibañez 1981) and is independent of the scales of the descriptors (Legendre and Legendre 1998). In the third step, the model calculates the probability of each grid point to belong to the reference matrix by using a simplified (i.e., by testing one observation instead of comparing a group of observations) version of the “Multiple Response Permutation Procedure” (MRPP; Mielke et al. 1981). This probability (*v*) is the number of times the simulated distance was found greater than or equal to the observed average distance: 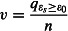
2with ε_0_ is the average observed distance, ε_*S*_ the recalculated distance after permutation, and *n* the maximum number of permutations. If the probability is close to 1, the environmental values of the tested point are at the center of the ecological niche. A probability close to zero indicates that the environmental conditions of the point are outside of the ecological niche. Finally, the last step consists in mapping the probability of species occurrence. This method was applied (1) to establish the ecological niche (*sensu* Hutchinson) of both species, (2) to model their spatial distribution for the baseline period (1950–2000), and (3) to project their future distribution using CMIP5 simulations (Moss et al. 2010). A high probability of occurrence corresponds to a region climatically suitable for the species.

### Model evaluation for baseline distribution

The performance of the model was assessed by applying the “Area Under the Curve” of the Receiver Operating Characteristic method (ROC). While the selection of a suitable procedure to evaluate presence-only models remains widely discussed in the literature (Peterson et al. 2008), the ROC curve method can be applied (Franklin 2009) by creating artificial absence data (usually termed pseudo-absence or background data; Phillips et al. 2006; Raybaud et al. 2013). The ROC plot is based on a series of misclassification matrices computed for a range of cutoffs from 0 to 1. It then plots on the *y*-axis the true positive rate (sensitivity) against the false-positive rate (1-specificity) from the same misclassification matrix (Fielding and Bell 1997; Pearce and Ferrier 2000). This procedure provides a value (the “Area Under the Curve” or AUC) representing the model accuracy. In the case of a presence-only model, the AUC value describes the probability that the model scores a random presence site higher than a random background site (Phillips et al., 2009) and the value varies between 0.5 (for random performance) and 1 (a perfect fit) (Brotons et al. 2004). We used a cross-validation procedure, as recommended by Merow et al. (2013) and performed by Tittensor et al. (2009), selecting 70% of data to run the model NPPEN and 30% to evaluate its performance. To investigate whether the random selection of data could influence the modelling of the ecological niche, five runs were performed for each species using different 70% random sample of the observed data (Table S5). Background locations (the grid cells without species presence; Phillips et al., 2006; Tittensor et al. 2009) were chosen randomly 500 times in the whole spatial domain (28°N–76°N, 14°W–61°E) to provide both an average and a standard deviation of the AUC value (Table S5).

### Threshold criterion for accurate predictions in species distribution

Absence data are often difficult to obtain accurately (Hirzel et al. 2002; Jiménez-Valverde and Lobo 2007) and false absence data can have negative effects on ENMs (Jiménez-Valverde and Lobo 2007). Prediction methods that use only presence data tend, in general, to overestimate distributions due to the lack of absence data (Ferrier and Watson 1997; Engler et al. 2004). To prevent this potential issue, we calculated the “Minimized Difference Threshold” (MDT criterion; Jiménez-Valverde and Lobo 2007) above which species are more likely to be present by minimizing the difference between both sensitivity and specificity obtained from the AUC method. This method is known to produce better predictions by removing false-positive presence (Liu et al. 2005; Jiménez-Valverde and Lobo 2007). Such an approach is required when the influence of climate change on species range is estimated (Liu et al. 2005).

### Projections of the spatial changes in species distribution

For each 20-year period from 2010–2029 to 2080–2099, we estimated the occurrence of *B. nana* and *C. sativa* by applying the NPPEN model based on environmental data originating from the seven GCMs and the four RCP scenarios (i.e., 25 simulations). First, we calculated for each species and simulation the proportion (as percentage) of the studied area (Europe, 28°N–76°N,14°W–61°E) that was projected to contain a suitable habitat. Second, for a given RCP, the seven GCMs were averaged to create an ensemble (a consensus among GCMs; Beaumont et al. 2008), which was subsequently used to project species distributions and determine the percentages of species occurrence. The percentages of species occurrence calculated from the ensemble were then compared to the percentages obtained for each simulation.

Between-GCM and between-RCP variability associated with the percentages of occurrence of the two species were estimated for the periods 2010–2029 and 2080–2099 by means of the coefficients of variation. The 95% confidence interval of each coefficient of variation was assessed by applying a normalized bootstrap (Davison and Hinkley 1997).

On the basis of the multi-GCM and multi-RCP approach, we then divided the 25 simulations into three projected species trends, based on the 33rd and 66th percentiles to evaluate the potential future distribution of *B. nana* and *C. sativa*: (1) pessimistic species trends (the negative extreme projections; 0–33rd percentile); (2) moderate species trends (the most common projections; 33–66th percentile); (3) optimistic species trends (the positive extreme projections; 66–100th percentile). For each projected species trend and geographical cell of the spatial domain, we calculated mean probabilities of occurrence. This average was based on the probabilities of occurrence retained after application of the MDT criterion (Table S5). We subsequently represented the future potential distributions of *B. nana* and *C. sativa* for the periods 2010–2029 and 2080–2099 and also mapped the coefficient of variation.

## Results

We compared both modeled baseline (1950–2000) distributions and gridded presence data from the *Atlas Florae Europaeae* (Fig.1). The accuracy of the projections assessed with the AUC statistics was high (AUC values of 0.80 ± 0.01 for *B. nana* and 0.88 ± 0.01 for *C. sativa*; Table S5). Both the Scandinavian distribution of *B. nana* and the western European range of *C. sativa* were well reproduced. The probability of occurrence of *B. nana* north of the Alps was, however, slightly lower than expected (Fig.1A,C), a feature that may be related to the consideration of air rather than soil temperatures (Pellissier et al. 2013). This bias was already observed for low-stature plants that may be decoupled from atmospheric conditions in regions with differential angles of solar radiation or with complex topography (Pellissier et al. 2013). In contrast, although a suitable habitat for *B. nana* was revealed in the Caucasus, the species does not occur in this area because the shrub requires high concentration of organic carbon typically found in taiga soils (Gundelwein et al. 2007). The presence of *C. sativa* detected along the southern coast of the Black Sea and in the Caucasus (Fig.1D) was corroborated by the distribution map compiled by the European Forest Genetic Resources Programme (http://www.euforgen.org; Fig. S1). Similarly, the modeled distribution in North Africa was substantiated by the literature (Haltofová and Jankovský, 2003; Krebs et al. 2004). In contrast, false-positive occurrences of *C. sativa* located in eastern Spain and in northwestern Europe may be explained by an unsuitable soil (i.e., alkaline and podzolic soils; Rubio et al. 2002).

**Figure 2:**
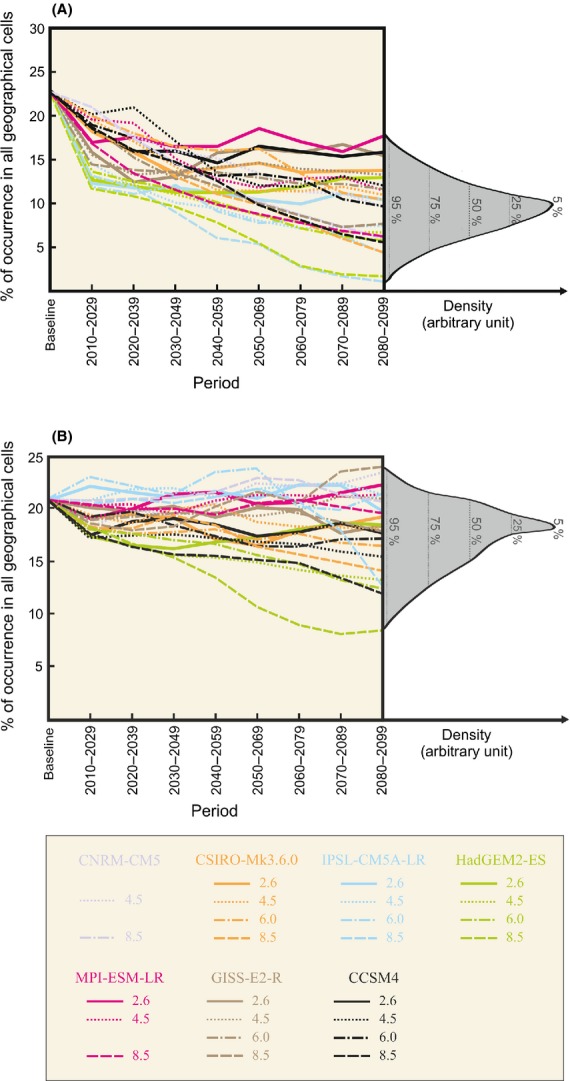
Long-term projected changes in the spatial extent (as percentage of occurrence) of (A) *Betula nana* and (B) *Castanea sativa* for each 20-year period of the 21st century, different intensities of warming and seven GCMs. Density diagrams (right) show the range of the percentages of occurrence for 2080–2099. Dotted vertical lines represent the percentiles 5%, 25%, 50%, 75%, and 95% of the distribution. The baseline period is 1950–2000. Percentages of occurrence were calculated for all climate scenarios (the low RCP2.6, the medium–low RCP4.5, the medium–high RCP6.0, and the high RCP8.5) and the seven GCMs: CNRM-CM5 (violet), CSIRO-Mk3.6.0 (orange), IPSL-CM5-LR (blue), HadGEM2-ES (green), MPI-ESM-LR (pink), GISS-ES-R (brown), and CCSM4 (black). The line-style denotes RCP climate scenarios. No simulations were available for the CNRM-CM5 with both RCP2.6 and RCP6.0 and for the MPI-ESM-LR with RCP6.0. See Table S4 for the meaning of GCMs.

The influence of climate change on the spatial distribution of the two species from the baseline period 1950–2000 to the end of this century was evaluated using data from seven GCMs and four emissions scenarios. In all geographical cells, the percentage of species occurrence was determined for both the baseline period and each 20-year period of the 21st century (Fig.2). Although some projections display a constant or a slight increase in the coverage of *C. sativa* (Fig.2B), most exhibit a long-term reduction in the spatial extent of the two species, the pattern being more pronounced for *B. nana* (Fig.2A). The analysis also reveals that future changes in the spatial coverage of the two species not only depend on the level of warming but also on GCMs. This is particularly apparent for *C. sativa* (e.g., model HadGEM2-ES versus IPSL-CM5A-LR). For this species, the difference between GCMs can even be higher than the difference between RCPs (e.g., GISS-E2-R Scenario 2.6 vs. Scenario 8.5). The phenomenon was less evident for *B. nana*. Although the pattern was also detected for the beginning of the 21st century (e.g., model CCSM4 versus HadGEM2-ES), it was less obvious at the end of the time period.

**Figure 3:**
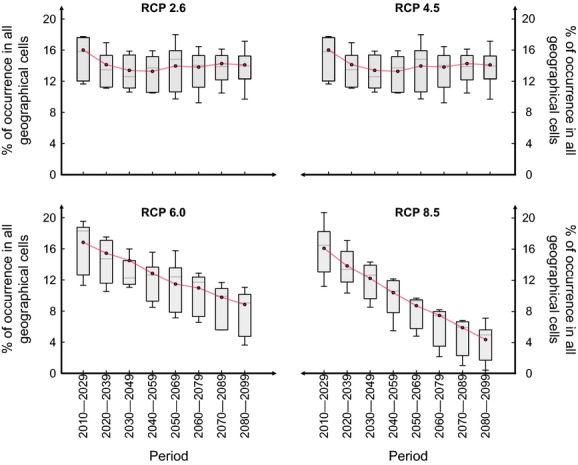
Comparison between changes in the spatial extent (as percentage of occurrence) of *Betula nana* calculated from the individual projections (boxplots in gray obtained from the different trajectories; Fig.2) and from averaging GCM outputs (red dotted-lines) for each 20-year period of the 21st century and each RCP scenario: the low RCP2.6, the medium–low RCP4.5, the medium–high RCP6.0, and the high RCP8.5.

**Figure 4:**
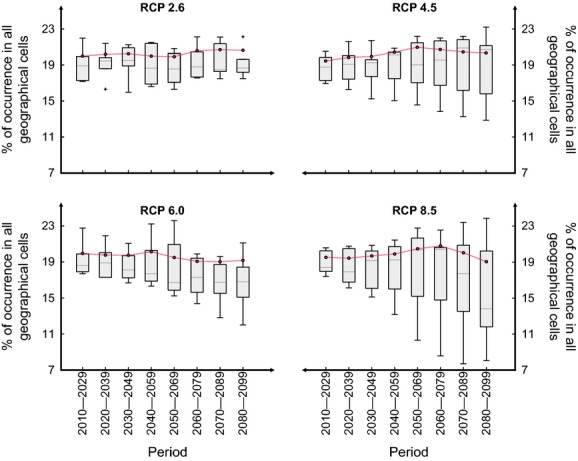
Comparison between changes in the spatial extent (as percentage of occurrence) of *Castanea sativa* calculated from the individual projections (boxplots in gray obtained from the different trajectories; Fig.2) and from averaging GCM outputs (red dotted-lines) for each 20-year period of the 21st century and each RCP scenario: the low RCP2.6, the medium–low RCP4.5, the medium–high RCP6.0, and the high RCP8.5.

To compare the range of potential trajectories associated with the projections and those calculated from the average of climate values (consensus among GCMs; Figs.3 and 4), we showed the between-GCM variability for each RCP scenario (RCP2.6 to RCP8.5) by means of boxplot (in gray) and superimposed the percentages of species occurrence obtained from climate scenario averages (in red). For *B. nana*, our results revealed that percentages of species occurrence associated with the consensus approach were closed to the median values of the range of potential trajectories (Fig.3). For *C. sativa*, percentages of species occurrence calculated from the average of the climate values were always greater than the median values, whatever the level of warming (Fig.4). The potential alteration in the percentages of *C. sativa* occurrence projected for high levels of warming was not visible (Fig.4). These comparisons show that the effects of extreme scenarios are masked when considering a consensus among GCMs instead of the individual projections from each GCM.

**Figure 5:**
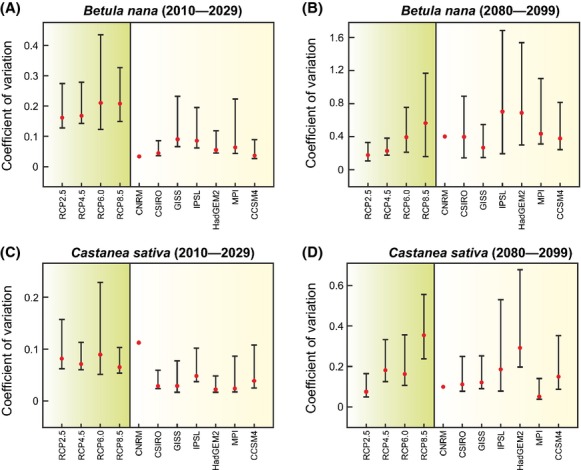
Quantification of the between-GCM (green) and between-RCP (yellow) variability in the percentages of species occurrence for *Betula nana* in (A) 2010–2029 and (B) 2080–2099 and *Castanea sativa* in (C) 2010–2029 and (D) 2080–2099. The red circles denote the coefficients of variation, and the black lines indicate the 95% confidence intervals estimated by bootstrap. See Figure2 and Table S4 for the meaning of GCMs.

We then quantified the between-GCM and between-RCP variability (as coefficients of variation) of projected spatial distribution of both *B. nana* and *C. sativa* for the period 2010–2029 and 2080–2099. During the first period, the between-GCM variability was greater than the between-RCP variability for both species and all scenarios (Fig.5A,C). The pattern was slightly stronger for *B. nana* than *C. sativa*. By the end of this century, both types of variability reached a similar level. However, values of the coefficients of variation characterizing the between-GCM variability for both species magnified when the radiative forcing increased (from RCP2.6 to RCP8.5; Fig.5B,D); the higher the radiative forcing was, the greater the between-GCM variability was. For Scenario 8.5, between-GCM variability for *C. sativa* was greater than the between-RCP variability (Fig.5D). For *B. nana*, between-GCM variability was also elevated, but slightly smaller than the between-RCP variability in the models IPSL-CM5A-LR and HadGEM2-ES (Fig.5B).

Based on our multi-GCM and multi-RCP approach, simulations were assigned into three projected species trends: optimistic, moderate, and pessimistic (Table S6). This approach allowed us to characterize distributional changes based on trajectories of greenhouse gas concentrations (RCPs) and projected spatial changes of species in response to environmental modifications. For the period 2080–2099, pessimistic trends for both species were mainly related to high emission scenarios (RCP6.0 and RCP8.5; Table S6) while optimistic trends were mostly associated with low emission scenarios (RCP2.6 and RCP4.5; Table S6). However, changes in species coverage were not always linearly related to the intensity of emission scenarios (“cascade of uncertainty” effect; Wilby and Dessai 2010) and simulations under high emissions scenarios sometimes led to optimistic trends in species coverage (Table S6).

**Figure 6:**
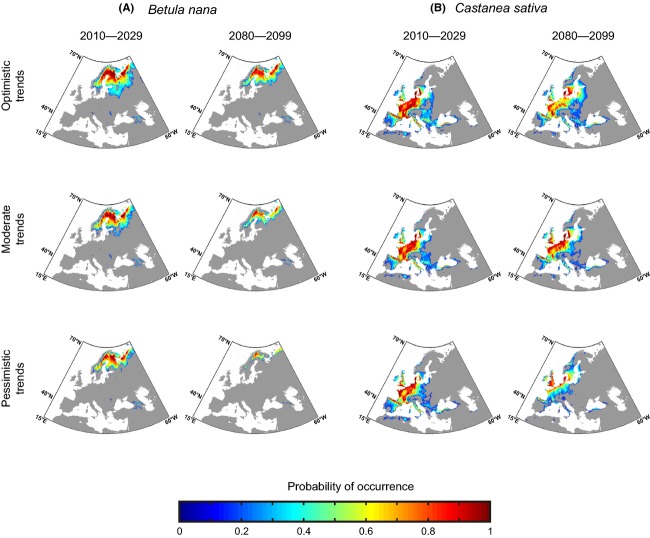
Projections of short-term (2010–2029) and long-term (2080–2099) changes in the spatial distribution of the averaged probability of occurrence of (A) *Betula nana* and (B) *Castanea sativa* for three projected species trends: pessimistic, moderate, and optimistic. These categories of projections were based on 25 runs (see Fig.2). Gray geographical cells denote probabilities under the MDT criterion (see Materials and Methods).

The difference between optimistic and pessimistic projected trends was more evident for *B. nana* than *C. sativa* for the period 2010–2029 (Fig.6). However at the end of this century (2080–2099), the dissimilarity between optimistic and pessimistic trends becomes substantial for the two species. Indeed, a reduction in the size of suitable climatic habitat of *B. nana* is expected and only some populations are likely to persist in Scandinavia (Fig.6A). In optimistic trends, an increase in the probability of occurrence of *C. sativa* was observed in regions located at the eastern and northern limits of its distribution. For pessimistic trends, alterations in the probability of occurrence of *C. sativa* took place at the southern limit of the spatial distribution and we observed a poleward biogeographic movement of the core region (i.e., geographical cells with the highest probabilities) toward the northwestern coast of Europe (Fig.6B).

## Discussion

At each step of a modelling procedure, several sources of variation that may contribute to the emergence of uncertainties exist (Beaumont et al. 2008). Characterizing the variability related to both ENMs and GCMs is likely to improve our perception of the sources of uncertainties in simulations of future species distributions (Beaumont et al. 2007; Beale and Lennon 2012). Although several studies evaluated the influence of warming on projections of future spatial distributions made by ENMs (Thuiller 2004; Alkemade et al. 2011; Cheaib et al. 2012), our study shows that in addition to this variability, a large and often underestimated source of uncertainty is also related to the existence of different GCMs (Beaumont et al. 2007; Buisson et al. 2010; Real et al. 2010). Although sometimes discussed in the literature (e.g., Wiens et al. 2009; Beale and Lennon 2012), this source of variability remains seldom quantified because it is difficult to fully explore uncertainties arising from GCMs (Diniz-Filho et al. 2009). Using a combined multi-GCM and multi-RCP approach, we show that a major part of the variability in projections is related to GCMs with a variability frequently as high as the warming intensity itself. The between-GCM variability was higher than the between-RCP variability for the next decades, and both types of variability reached a similar level at the end of the century. This result reveals that discrepancies between projections may be more attributable to GCMs themselves than to the presumed effect of different radiative forcing (Real et al. 2010). We provide evidence that both the choice of GCMs and scenarios could greatly influence the projections of future species distributions and that the use of different GCMs may lead to conflicting projected distributional ranges of species (Xu and Yan 2001). In addition, our analyses also show that the between-GCM variability increases with the intensity of warming, with uncertainties increasing through time, probably due to the nonlinear nature of the climate system (Beaumont et al. 2007).

Our results show that regions climatically suitable for the two European species *B. nana* and *C. sativa* are likely to be altered by global warming in the next decades. As already observed in the dynamics of biogeographic ranges in terrestrial and marine realms (Gaston 2003; Beaugrand et al. 2011), the variability from optimistic to moderate and pessimistic projected species trends were more pronounced at the edge of the spatial distributions of species, whereas their centers were less variable (Fig. S6). Although these species are long-lived and may persist for years after environmental conditions become unsuitable (Matthews et al. 2011), species unable to track their environmental envelope could lose a significant amount of their habitats (Lenoir et al. 2011). Biogeographic species movements and local extirpation may have major impacts on the functioning of ecosystems and the services they provide (Pereira et al. 2010; Hanewinkel et al. 2013). For example, change in the distribution of *B. nana* (as expected under pessimistic trends) could have severe consequences for the subarctic climate (Sturm et al. 2001a) and the amount of carbon stored in soils (Sturm et al. 2001b). These alterations may not only be directly caused by physiological stress, but also indirectly via an imbalance of species interactions (Allen et al. 2010; Northfield and Ives 2013).

One of the main goals of modelling and projecting species distributional ranges is to inform decision-makers on the potential implications of climate change on species by providing a range of alternative futures. This projecting approach uses future environmental conditions estimated according to the combination of GCMs and scenarios on forecasted radiative forcing (i.e., the balance between incoming and outgoing radiation; Moss et al. 2010). Despite potential shortcomings inherent to the application of the ecological niche in biogeographic research (Pearson and Dawson 2003; Araújo and Peterson 2012), ENMs represent valuable and cost-effective tools to determine potential changes in species distribution in the context of global warming, especially in poorly monitored regions (Marmion et al. 2009).

In our study, we chose to focus on uncertainties caused by GCMs (Real et al. 2010). GCMs may differ for a wide range of reasons (Beaumont et al. 2008). For example, each integrates distinct algorithms to portray the dynamics of atmospheric circulation and to model feedbacks between the land/ocean surface and the atmosphere (Wiens et al. 2009). To date, no criterion exists to evaluate GCMs (Fordham et al. 2011) and their performance may vary among variables and regions (e.g., Fordham et al. 2011). Therefore, applying multi-GCM and multiscenario approaches in ecological niche modelling enables the consideration of a range of possible futures. While uncertainty is intrinsic to the climate system and cannot be avoided, identifying and quantifying sources of variation is an important prerequisite (Beaumont et al. 2008). Considering several climate models in the analysis is important to improve our understanding of the degree of uncertainty on projections of future species distribution (Weaver and Zwiers 2000; Wiens et al. 2009). While earlier studies noted that at least five GCMs are required in such approaches (Perkins et al. 2009; Pierce et al. 2009; Fordham et al. 2011), our results highlight that it is better to use a large number of GCMs (Laepple et al. 2008; Naujokaitis-Lewis et al. 2013).

In the recent past, a majority of studies (more than 70% of articles published between 2008 and 2010; Fordham et al. 2011) relied on a single GCM to project the effects of climate change on future species distributions, masking a considerable source of uncertainty (Fordham et al. 2011). As a way to account for uncertainties related to different GCMs, developing consensus among GCMs (i.e., averaging climate models) has been proposed by ecologists (Araújo and New 2007; Fordham et al. 2011). Such an approach could, however, present potential biases depending, for example, on the GCMs retained for the creation of ensembles (Buisson et al. 2010; Garcia et al. 2012; Naujokaitis-Lewis et al. 2013). By comparing simulations obtained from each GCM with those obtained from an average of climate models, our results confirmed that climate scenario ensembles mask potential trajectories associated with GCM outputs. The use of ensembles by deriving the central tendency of forecasts (Araújo and New 2007; Pierce et al. 2009; Garcia et al. 2012) leads to a loss of variability, the effects of extreme scenarios being masked (Beaumont et al. 2008; Naujokaitis-Lewis et al. 2013). More importantly, consensus among GCMs may not reflect an observable climatic state. This was well summarized by Beaumont et al. (2008) who wrote “*a system that is either very wet (1.0) or very dry (0.0) will have an average (0.5) that does not exist in nature*”.

Consensus techniques are therefore unlikely to provide accurate estimates of climate change impacts on future species distributions (Naujokaitis-Lewis et al. 2013) and averaging the simulations may instead hide uncertainties (Beaumont et al. 2007) even if hybrid consensus approaches attenuate possible caveats (Garcia et al. 2012). By definition, the future is uncertain (Wiens et al. 2009) and this is why each realization should be examined rather than averaged (Beaumont et al. 2007; Beale and Lennon 2012) to provide the full range of potential trajectories associated with the projections as well as to increase the relevance of ENM projections (Pereira et al. 2010; Parmesan et al. 2011). Although a multi-GCM approach will not remove all uncertainties, it makes their reporting more explicit (Beaumont et al. 2007) and enables exploration of the potential outcomes and highlights extremes (Naujokaitis-Lewis et al. 2013). For example, compared to other climate models, the HadGEM2-ES model projected an extreme reduction in the probability of occurrence of *B. nana* and *C. sativa* by the end of the 21st century, a feature already observed for needleleaf species in Europe (Betts et al. 2013).

If nothing is done to alleviate global warming, future species spatial distribution may become more and more difficult to anticipate. In a changing world, improving the reliability of species projections is what managers and conservationists expect from scientists (Dawson et al. 2011). How should uncertainty be treated to provide more realistic ecological scenarios? Based on our results, we propose to use multi-GCM and multi-emission scenario approaches to better anticipate potential trajectories and quantify uncertainties in projected species distributions. Density diagrams (Fig.2), which display all potential trajectories, allow the most common signal as well as extreme trends (optimistic and pessimistic) to be identified. Presenting the median and range of potential changes in species distribution is known to provide more information than consensus procedures (Beaumont et al. 2007). However, we acknowledge that such a method may lead to computational limitations when very large sets of species are considered and alternative/complementary approaches may be adopted. For instance, performing multivariate techniques to group similar GCMs may prove efficient (e.g., principal component analysis, Thuiller 2004; clustering methods, Garcia et al. 2012). Beaumont et al. (2007) suggest projecting potential species distributions by constructing probabilistic climate change projections based on several GCM realizations/simulations (Dessai et al. 2005). GCM-performance ranking techniques allow giving less confidence to GCMs for which future climate conditions are considered unreliable and may also be a way to reduce the between-GCM variability (Macadam et al. 2010). However, an important consideration is that GCM performance can be assessed only relative to past observations and although some GCMs perform better than others, no individual GCM undoubtedly emerges as “the best” overall (Fordham et al. 2011; IPCC 2013). This issue has been widely addressed in the last IPCC Report (IPCC 2013; see Chapter 9). Climate models, based on physical principles, are able to reproduce many important aspects of past response to external forcing and climate predictions can be regularly verified. Climate projections spanning a century cannot (IPCC 2013). This is particularly the case as anthropogenic radiative forcing may drive the climate system toward conditions not previously observed in the instrumental record (IPCC 2013).

## Supporting Information

**Figure S1.** Distribution map of the occurrence of *Castanea sativa* in Europe from the European Forest Genetic Resources Programme (http://www.euforgen.org). **Figure S2.** Results from the Escoufier procedure (See Materials and Methods). **Figure S3.** Modelled ecological niche of *Betula nana* assessed from NPPEN and based on four environmental factors represented by pairs. **Figure S4.** Modelled ecological niche of *Castanea sativa* assessed from NPPEN and based on five environmental factors represented by pairs. **Figure S5.** Description of the ‘delta method’ procedure. **Figure S6.** Variability in the probability of occurrence of (A) *Betula nana* and (B) *Castanea sativa* for the periods 2010–2029 and 2080–2099 and three projected species trends. **Table S1.** Environmental data retrieved from the WorldClim dataset. **Table S2.** Environmental parameters retained from the WorldClim dataset (Table S1) after application of the Escoufier procedure (See Materials and Methods). **Table S3.** The four RCPs and main similarities and differences between temperature projections for SRES and RCPs. From Moss et al. and Rogelj et al.). **Table S4.** General Circulation Models (GCMs) used in this study. **Table S5.** Minimised Difference Threshold (“MDT”) criterion and statistical summary of AUC values from the ROC curve procedure; average (Mean), minimum (Min), maximum (Max) and standard deviation (SD) of the AUC value for each species. **Table S6.** Table of the 25 simulations distributed within the three projected trends (pessimistic, moderate and optimistic) for both species and the two periods 2010–2029 and 2080–2099.
