# Supplementary material for: Uncertainties in the projection of species distributions related to general circulation models
Source: Ecol Evol. 2015 Feb 13;5(5):1100–16. doi: 10.1002/ece3.1411 (PMC4364824; doi:10.1002/ece3.1411)
Supplement: Supplementary file 1 [file ece30005-1100-sd1.docx]

**Supporting Information**

**Uncertainties in the projection of species distributions related to general circulation models**

Eric Goberville^1,2,3*^, Grégory Beaugrand^2,3,4^, Nina-Coralie Hautekèete^1^, Yves Piquot^1^ and Christophe Luczak^3,5,6^

^1^ Université Lille 1 - Sciences et Technologies (USTL), UMR 8198 GEPV, Laboratoire de Génétique et Evolution des Populations Végétales, F-59655 Villeneuve d'Ascq, FR

^2^ Université Lille 1 - Sciences et Technologies (USTL), UMR 8187 LOG, Laboratoire d’Océanologie et de Géosciences, 28 Avenue Foch, F-62930 Wimereux, FR

^3^ CNRS, UMR 8187 LOG, Laboratoire d’Océanologie et de Géosciences, 28 Avenue Foch, F-62930 Wimereux, FR

^4^ SAHFOS (Sir Alister Hardy Foundation for Ocean Science), The Laboratory, Citadel Hill, Plymouth PL1 2PB, UK

^5^ Université d’Artois, ESPE, Centre de Gravelines, 40 rue Victor Hugo - BP 129, 59820 Gravelines, FR

^6^ Université Lille Nord de France, FR

* To whom correspondence should be addressed:

E-mail: [eric.goberville@univ-lille1.fr](mailto:eric.goberville@univ-lille1.fr)

**Submitted to**

**Ecology and Evolution**

**Supplementary Figures and Tables**





**Table S1.** Environmental data retrieved from the WorldClim dataset. Variables are derived from the monthly temperature and rainfall values in order to generate more biologically meaningful variables. The variables represent annual trends (e.g. mean annual temperature, annual precipitation…), seasonality (as standard deviation for temperature and coefficient of variation for precipitation) and extreme or limiting environmental factors. A quarter is a period of three months (1/4 of the year). They are coded from 1 to 19 following the indications on the WorldClim website (http://www.worldclim.org/).


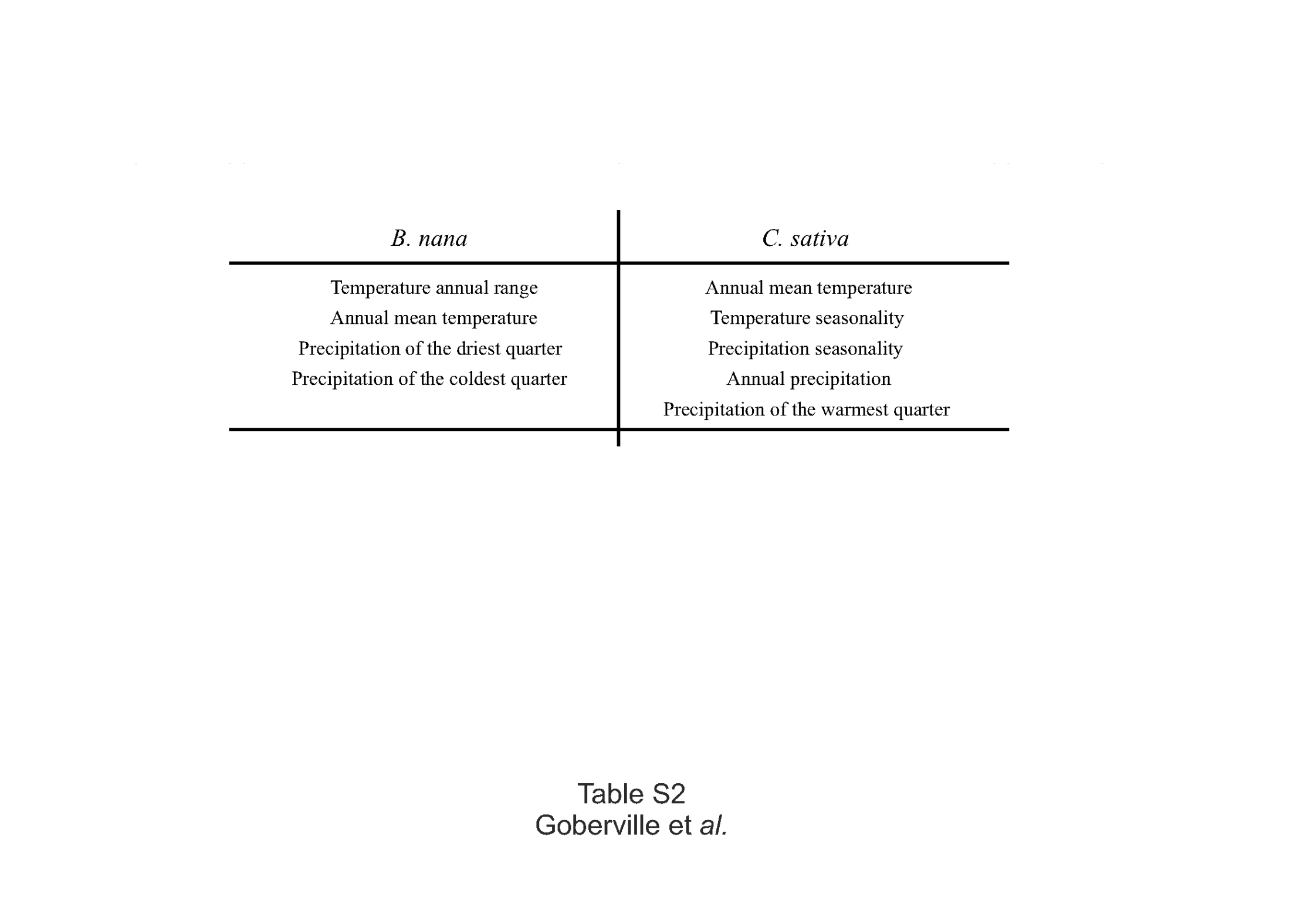


**Table S2.** Environmental parameters retained from the WorldClim dataset (Table S1) after application of the Escoufier procedure (See Materials and Methods).

**Table S3.** The four RCPs and main similarities and differences between temperature projections for SRES and RCPs. From Moss *et al.*, (2010) and Rogelj *et al.*, (2012).


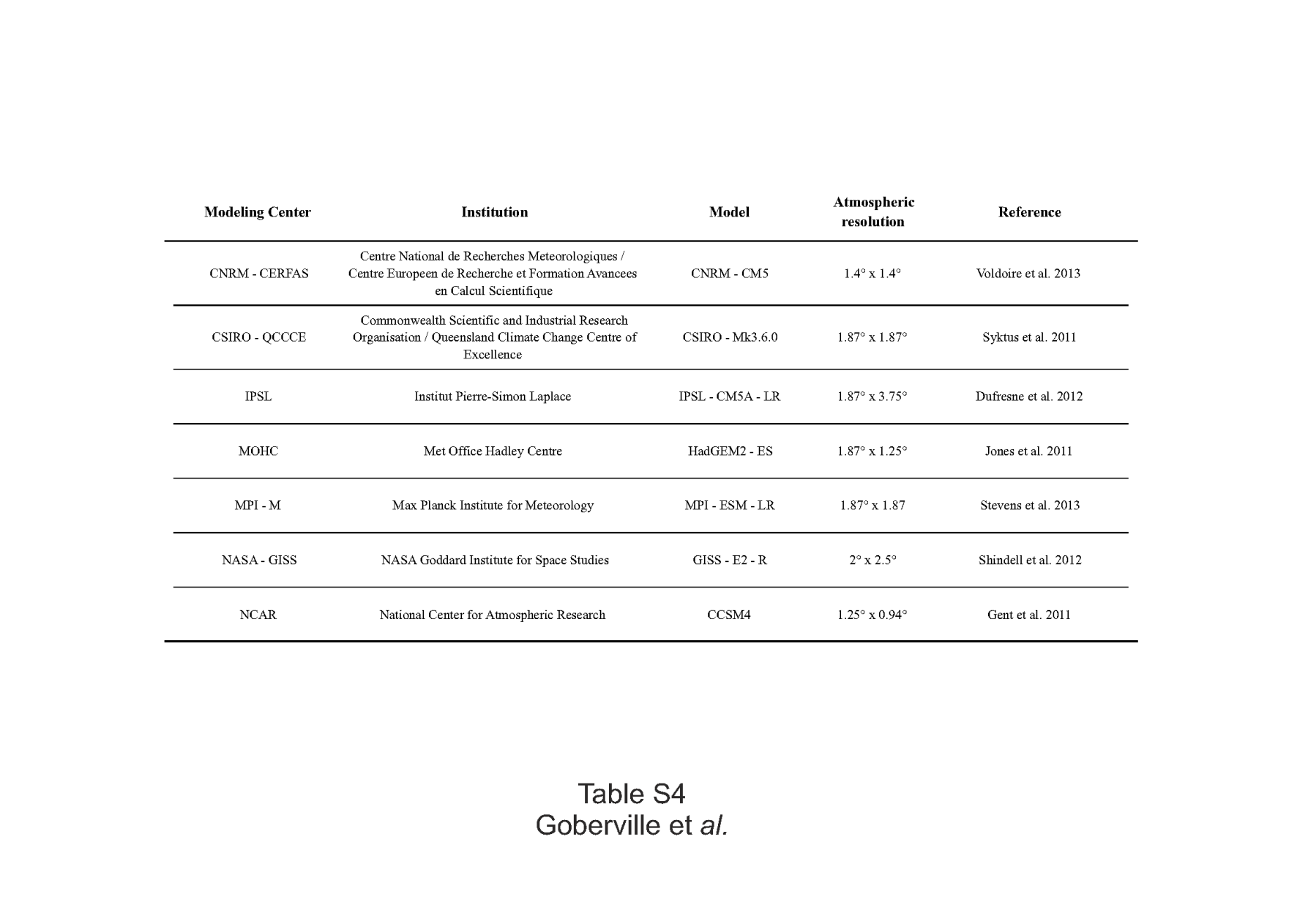


**Table S4.** General Circulation Models (GCMs) used in this study. Detailed descriptions of the different GCMs can be found in the references cited in the Table.

**Table S5.** Minimised Difference Threshold (‘MDT’) criterion and statistical summary of AUC values from the ROC curve procedure; average (Mean), minimum (Min), maximum (Max) and standard deviation (SD) of the AUC value for each species. Five model runs were performed for each species where a different random partitioning of the occurrence data (70% for calibration and 30% for validation) was used at each time.

**Table S6.** Table of the 25 simulations distributed within the three projected trends (pessimistic, moderate and optimistic) for both species and the two periods 2010-2029 and 2080-2099. See the Methods section ‘Projections of the spatial changes in species distribution’ for details on the categories. See Table S4 for the meaning of GCMs.


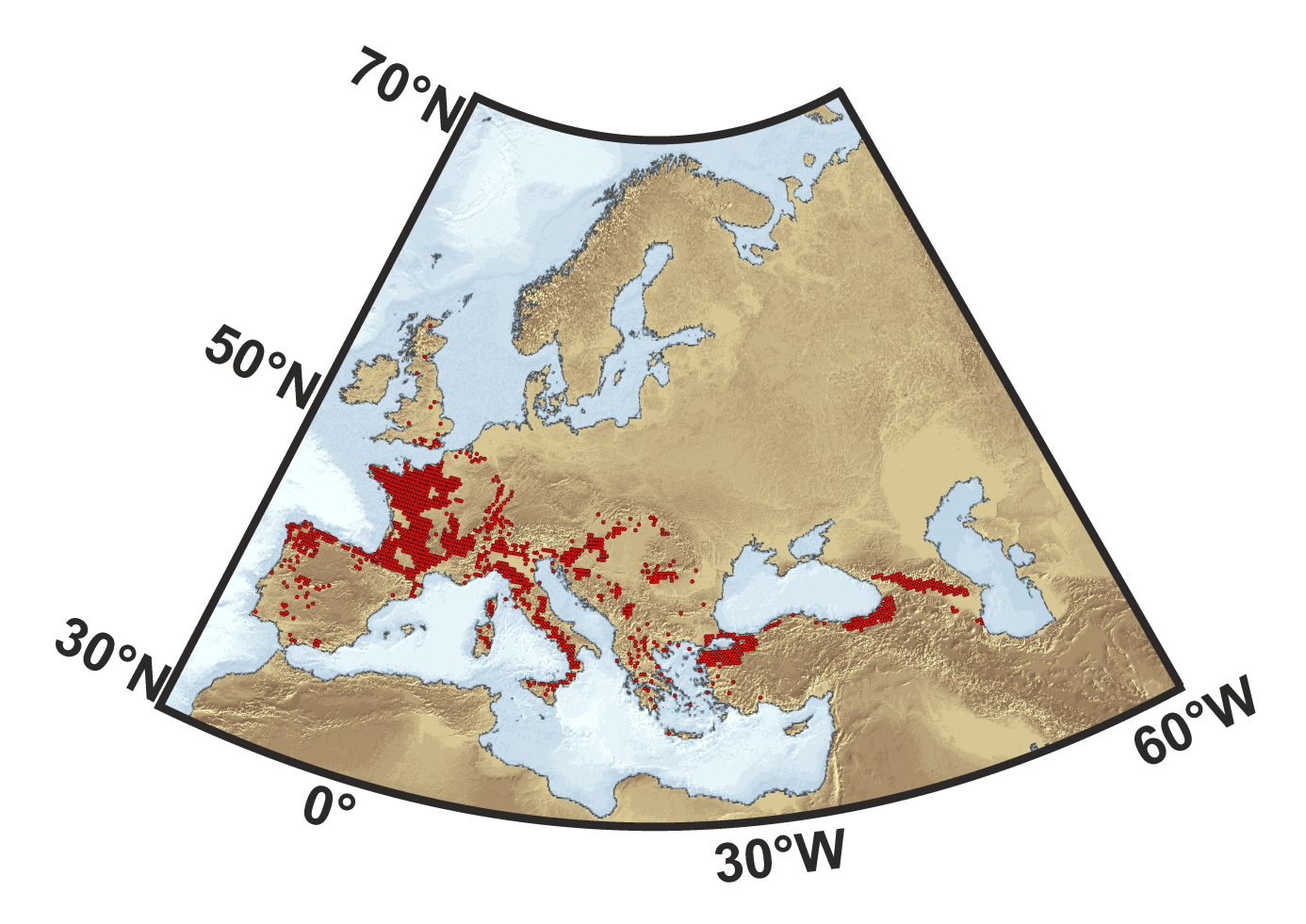


**Figure S1.** Distribution map of the occurrence of *Castanea sativa* in Europe from the European Forest Genetic Resources Programme (http://www.euforgen.org).


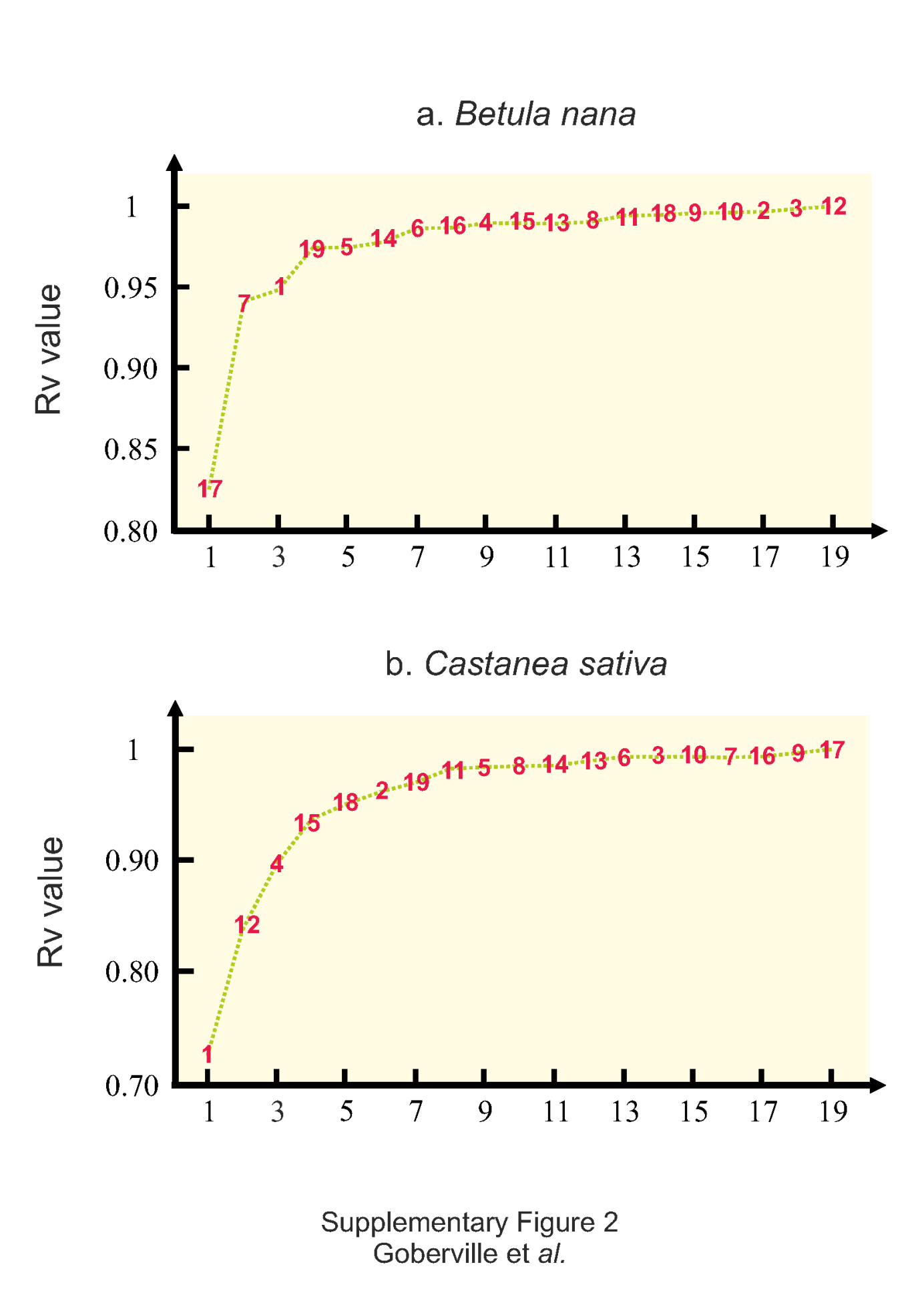


**Figure S2.** Results from the Escoufier procedure (See Materials and Methods). Environmental factors are coded from 1 to 19 following the indications on the WorldClim dataset (Table S1).


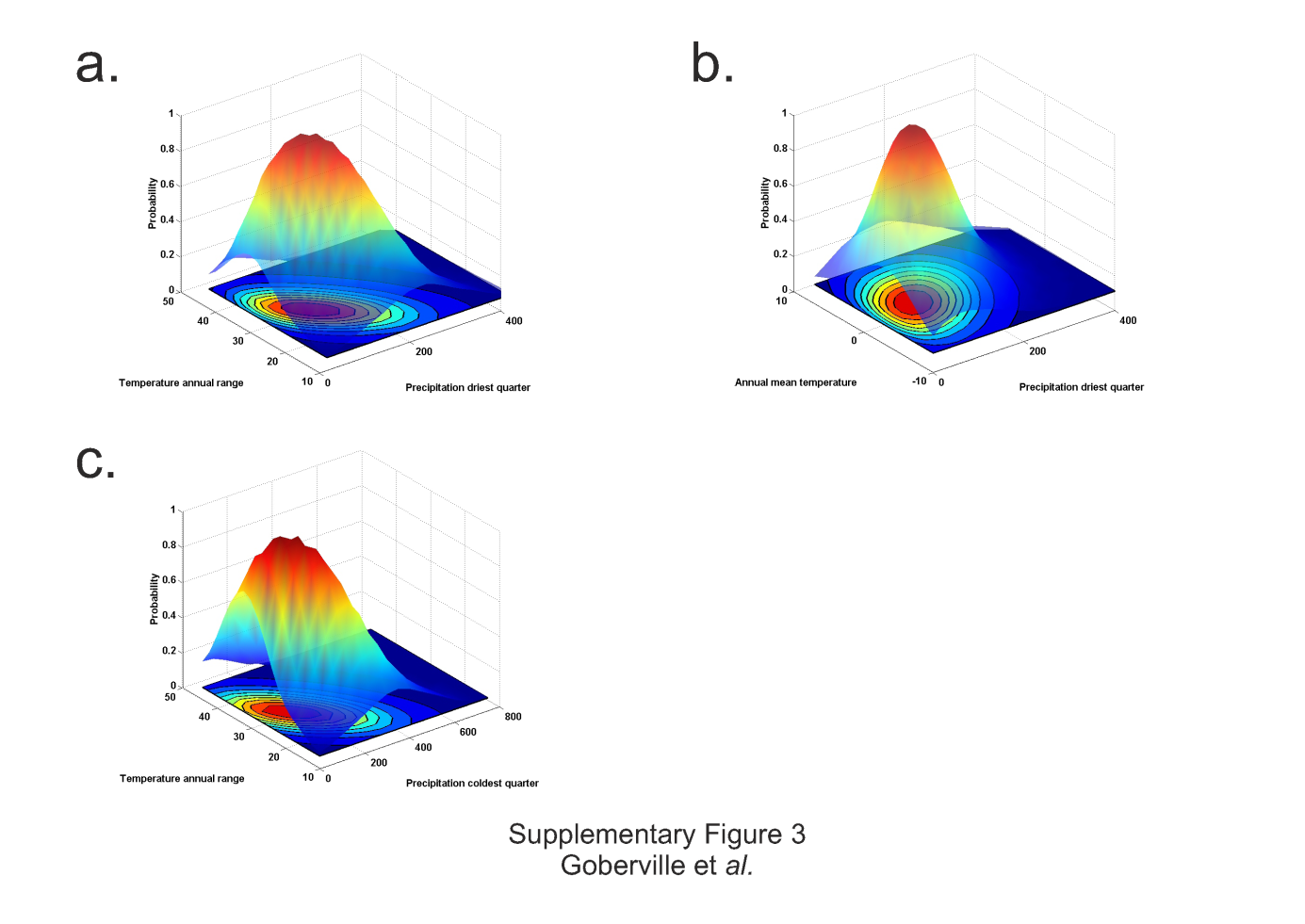


**Figure S3.** Modelled ecological niche of *Betula nana* assessed from NPPEN and based on four environmental factors represented by pairs. **a.** Precipitation of the driest quarter and Temperature annual range; **b.** Precipitation of the driest quarter and Annual mean temperature; **c.** Precipitation of the coldest quarter and Temperature annual range. The colorbar indicates the probability of occurrence of *B. nana*.


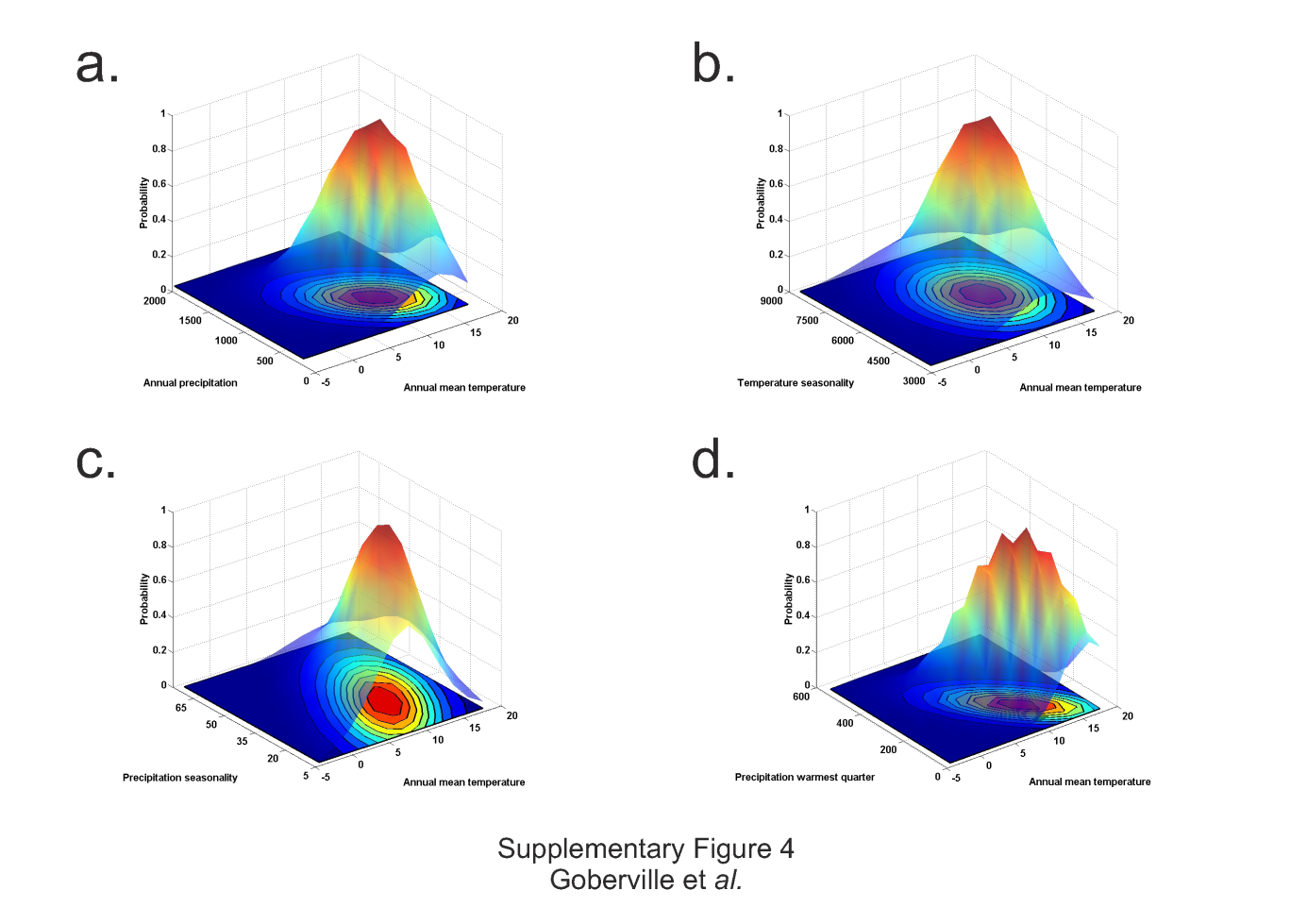


**Figure S4.** Modelled ecological niche of *Castanea sativa* assessed from NPPEN and based on five environmental factors represented by pairs. **a.** Annual mean precipitation and Annual mean temperature; **b.** Temperature seasonality and Annual mean temperature; **c.** Precipitation seasonality and Annual mean temperature; **d.** Precipitation of the warmest quarter and Annual mean temperature. The colorbar indicates the probability of occurrence of *C. sativa*.


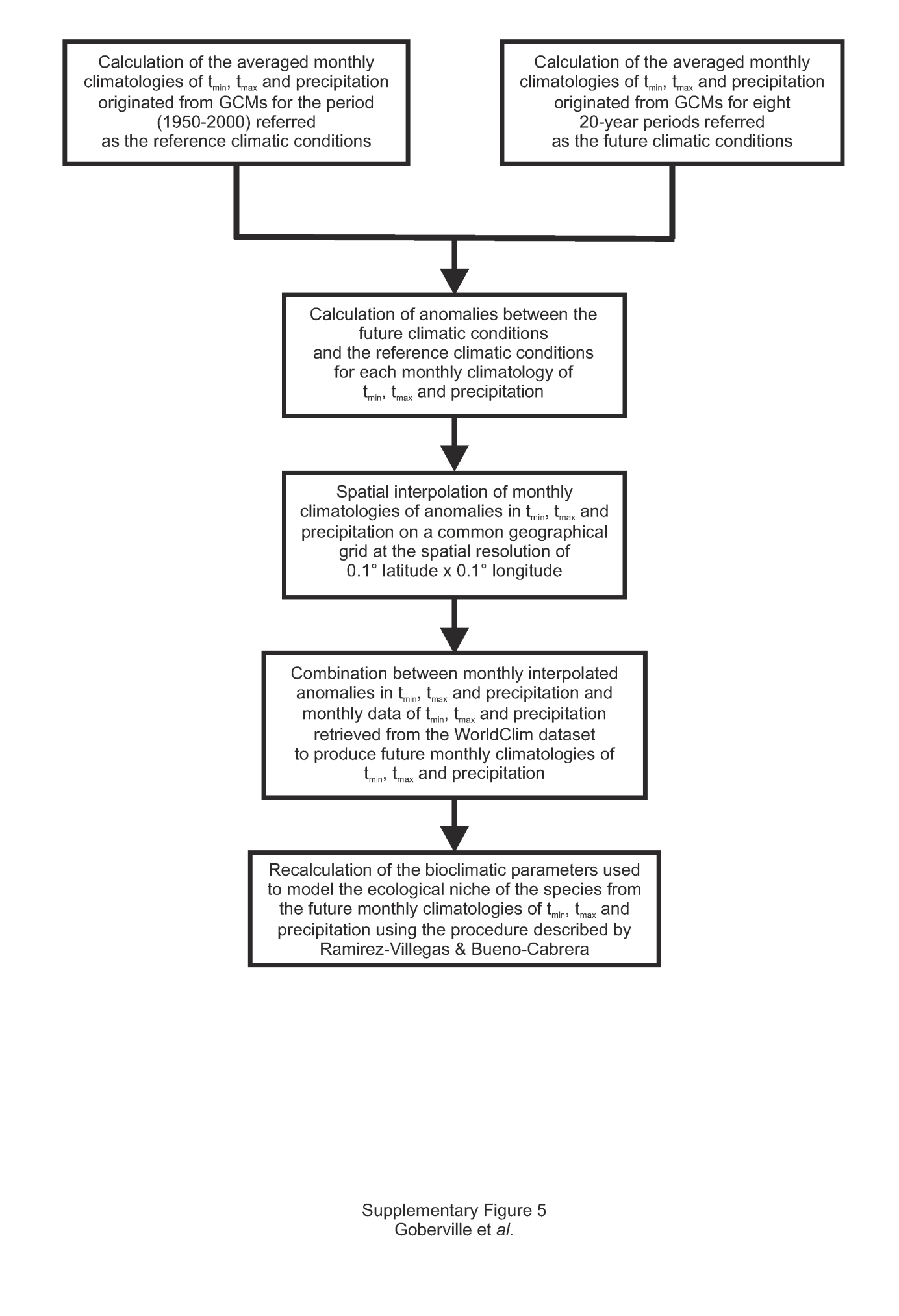


**Figure S5.** Description of the ‘delta method’ procedure. GCM: General Circulation Model, t_min_: temperature minimum, t_max_: temperature maximum. Adapted from Ramirez & Jarvis (2010).


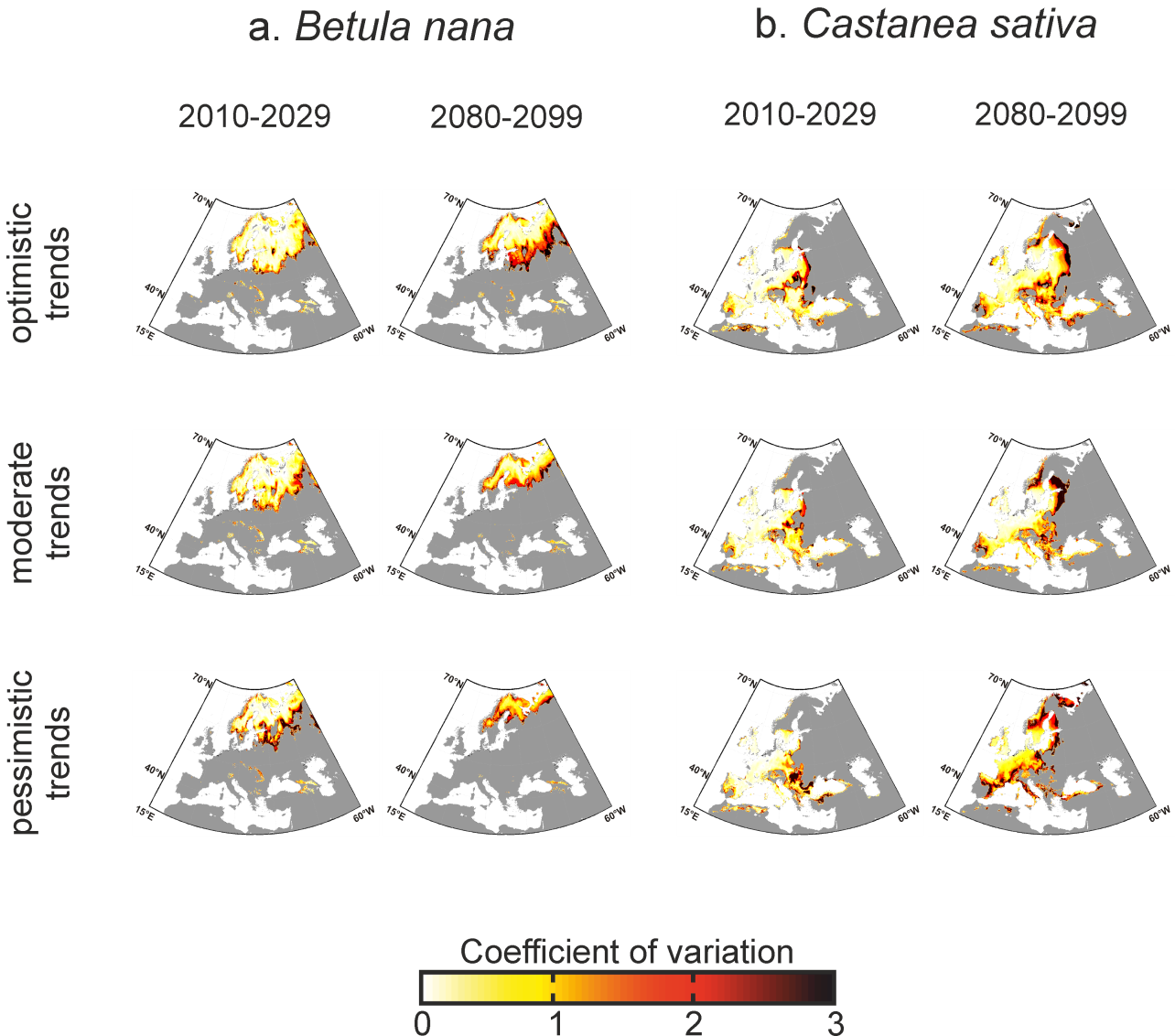


**Figure S6.** Variability in the probability of occurrence of a) *Betula nana* and b) *Castanea sativa* for the periods 2010-2029 and 2080-2099 and three projected species trends. A coefficient of variation was calculated for each geographical cell of our spatial domain, each trend (pessimistic, moderate and optimistic) and each time period. Grey geographical cells denote probabilities under the MDT criterion (see text).

**Supplementary references**

Dufresne, J.L., Foujols, M.A., Denvil, S., Caubel, A., Marti, O., Aumont, O., Balkanski, Y., Bekki, S., Bellenger, H., Benshila, R., Bony, S., Bopp, L., Braconnot, P., Brockmann, P., Cadule, P., Cheruy, F., Codron, F., Cozic, A., Cugnet, D., Noblet, N., Duvel, J.P., Ethé, C., Fairhead, L., Fichefet, T., Flavoni, S., Friedlingstein, P., Grandpeix, J.Y., Guez, L., Guilyardi, E., Hauglustaine, D., Hourdin, F., Idelkadi, A., Ghattas, J., Joussaume, S., Kageyama, M., Krinner, G., Labetoulle, S., Lahellec, A., Lefebvre, M. P., Lefevre, F., Levy, C., Li, Z.X., Lloyd, J., Lott, F., Madec, G., Mancip, M., Marchand, M., Masson, S., Meurdesoif, Y., Mignot, J., Musat, I., Parouty, S., Polcher, J., Rio, C., Schulz, M., Swingedouw, D., Szopa, S., Talandier, C., Terray, P., Viovy, N. & Vuichard, N. (2012) Climate change projections using the IPSL-CM5 Earth System Model: from CMIP3 to CMIP5. *Climate Dynamics*, **40**, 2123-2165.

Gent, P.R., Danabasoglu, G., Donner, L.J., Holland, M.M., Hunke, E.C., Jayne, S.R., Lawrence, D.M., Neale, R.B., Rasch, P.J., Vertenstein, M., Worley, P.H., Yang, Z.-L. & Zhang, M. (2011) The community climate system model version 4. *Journal of Climate*, **24**, 4973-4991.

Jones, C.D., Hughes, J.K., Bellouin, N., Hardiman, S.C., Jones, G.S., Knight, J., Liddicoat, S., Andres, R.J. & Bell, C. (2011) The HadGEM2-ES implementation of CMIP5 centennial simulations. *Geoscientific Model Development Discussion*, **4**, 689-763.

Moss, R.H., Edmonds, J.A., Hibbard, K.A., Manning, M.R., Rose, S.K., van Vuuren, D.P., Carter, T.R., Emori, S., Kainuma, M., Kram, T., Meehl, G.A., Mitchell, J.F.B., Nakicenovic, N., Riahi, K., Smith, S.J., Stouffer, R.J., Thomson, A.M., Weyant, J.P. & Wilbanks, T.J. (2010) The next generation of scenarios for climate change research and assessment. *Nature,* **463**, 747-756.

Rogelj, J., Meinshausen, M., Knutti, R. (2012) Global warming under old and new scenarios using IPCC climate sensitivity range estimates. *Nature Climate Change*, **2**, 248-253.

Shindell, D.T., Lamarque, J-F., Schulz, M., Flanner, M., Jiao, C., Chin, M., Young, P., Lee, Y.H., Rotstayn, L., Milly, G., Faluvegi, G., Balkanski, Y., Collins, W.J., Conley, A.J., Dalsoren, S., Easter, R., Ghan, S., Horowitz, L., Liu, X., Myhre, G., Nagashima, T., Naik, V., Rumbold, S., Skeie, R., Sudo, K., Szopa, S., Takemura, T., Voulgarakis, A. & Yoon, J-H. (2012) Radiative forcing in the ACCMIP historical and future climate simulations. *Atmospheric Chemistry and Physics Discussion*, **12**, 21105-21210.

Stevens, B., Giorgetta, M., Esch, M., Mauritsen, T., Crueger, T., Rast, S., Salzmann, M., Schmidt, H., Bader, J., Block, K., Brokopf, R., Fast, I., Kinne, S., Kornblueh, L., Lohmann, U., Pincus, R., Reichler, T. & Roeckner, E. (2013) Atmospheric component of the MPI-M earth System Model: ECHAM6. *Journal of Advances in Modeling Earth Systems*, **5**, 1-27.

Syktus, J., Jeffrey, S., Rotstayn, L., Wong, K., Toombs, N., Dravitzki, S., Collier, M., Hamalainen, C. & Moeseneder, C. (2011) *The CSIRO-QCCCE contribution to CMIP5 using the CSIRO Mk3.6 climate model*. (eds MODSIM) 2782-2788 (19th International Congress on Modelling and Simulation).

Voldoire, A., Sanchez-Gomez, E., Salas y Mélia, D., Decharme, B., Cassou, C., Sénési, S., Valcke, S., Beau, I., Alias, A., Chevallier, M., Déqué, M., Deshayes, J., Douville, H., Fernandez, E., Madec, G., Maisonnave, E., Moine, M.P., Planton, S., Saint-Martin, D., Szopa, S., Tyteca, S., Alkama, R., Belamari, S., Braun, A., Coquart, L. & Chauvin, F. (2013) The CNRM-CM5. 1 global climate model: description and basic evaluation. *Climate Dynamics*, **40**, 2091-2121.
